# Evolution of isoprene emission in Arecaceae (palms)

**DOI:** 10.1111/eva.13169

**Published:** 2020-12-14

**Authors:** Mingai Li, Jia Xu, Fuling Lyu, Iuliia Khomenko, Franco Biasioli, Mariacristina Villani, Barbara Baldan, Claudio Varotto

**Affiliations:** ^1^ Department of Biodiversity and Molecular Ecology, Research and Innovation Centre Fondazione Edmund Mach San Michele all'Adige Italy; ^2^ Experimental Center of Forestry in North China Chinese Academy of Forestry Beijing China; ^3^ Department of Food Quality and Nutrition, Research and Innovation Centre Fondazione Edmund Mach San Michele all'Adige Italy; ^4^ Botanical Garden of Padova University of Padova Padova Italy; ^5^ Department of Biology University of Padova Padova Italy

**Keywords:** Arecaceae, isoprene, isoprene synthase, IspS diagnostic tetrad, PTR‐ToF‐MS, site‐directed mutagenesis, transgenic Arabidopsis

## Abstract

Isoprene synthase (IspS) is the sole enzyme in plants responsible for the yearly emission in the atmosphere of thousands of tonnes of the natural hydrocarbon isoprene worldwide. Species of the monocotyledonous family Arecaceae (palms) are among the highest plant emitters, but to date no *IspS* gene from this family has been identified. Here, we screened with PTR‐ToF‐MS 18 genera of the Arecaceae for isoprene emission and found that the majority of the sampled species emits isoprene. Putative *IspS* genes from six different genera were sequenced and three of them were functionally characterized by heterologous overexpression in *Arabidopsis thaliana*, demonstrating that they encode functional IspS genes. Site‐directed mutagenesis and expression in Arabidopsis demonstrated the functional relevance of a novel IspS diagnostic tetrad from Arecaceae, whose most variable amino acids could not preserve catalytic function when substituted by a putatively dicotyledonous‐specific tetrad. In particular, mutation of threonine 479 likely impairs the open–closed transition of the enzyme by altering the network of hydrogen bonds between helices H1α, H, and I. These results shed new light on the evolution of IspS in monocots, suggesting that isoprene emission is an ancestral trait within the Arecaceae family. The identification of IspS from Arecaceae provides promising novel enzymes for the production of isoprene in heterologous systems and allows the screening and selection of commercially relevant palm varieties with lower environmental impact.

## 1 INTRODUCTION

Isoprene (2‐methyl‐1,3‐butadiene, C_5_H_8_) is a very abundant biogenic volatile compound, constituting about two thirds of all nonmethane biogenic volatile compounds (Guenther et al., 2006; Sindelarova et al., 2014). Isoprene is produced by organisms as diverse as bacteria, fungi, and algae, but large majority of this hydrocarbon is produced by land plants (McGenity et al., 2018). About 20% of plant species (Loreto & Fineschi, 2015), mainly perennial, fast‐growing forest tree species, naturally emit into the atmosphere large quantities of isoprene, corresponding to ~500 Tg C at global level annually (Dani et al., 2014; Guenther et al., 2012). Isoprene biosynthesis is catalyzed by the isoprene synthase (IspS) which in plants is nuclear‐encoded and targeted to chloroplasts where it uses as substrate the dimethylallyl diphosphate anion (DMADP) generated through the 2‐C‐methyl‐D‐erythritol 4‐phosphate (MEP) pathway (Schwender et al., 1997; Silver & Fall, 1995). Several studies in different plant species have shown that isoprene synthase is highly activated at temperatures around 40–45°C, pH 7.0–10.5, and generally in the presence of Mg^2+^ (Sasaki et al., 2005; Schnitzler et al., 2005; Silver & Fall, 1995). The generation of a crystal structure of PcISPS from poplar hybrid (*Populus x canescens* (Aiton) Sm.) identified important evidences for the metal‐binding motifs (the “aspartate‐rich” motif D345DXXD and the “NSE/DTE” motif N489DXXSXXXE) and the interaction sites F338, V341, and F485 in isoprene synthase active site pocket with substrate (Köksal et al., 2010). Following this protein structure model and the obtainment of newly identified isoprene synthase sequences, the amino acids F338, S446, F485, and N505 of PcISPS, corresponding to F310, S418, F457, and S477 in the IspS from the monocot *Arundo donax* (Li et al., 2017), were suggested as unique to isoprene synthases (Sharkey et al., 2013). Among these four sites, the two key sites F310 and F457 are considered as a marker to distinguish the isoprene synthases from other terpene synthases, and the other two sites are deemed important for isoprene synthases in general. Later on, these sites were commonly and successfully used to screen or identify novel isoprene synthases in other plant species, which is why they are normally referred to as the IspS diagnostic tetrad (Ilmén et al., 2015; Li et al., 2017). Although the conservation of these sites in isoprene synthases among different species from different families is often observed, the functional relevance of these sites for isoprene emission was demonstrated thoroughly by site‐specific mutagenesis only for the two residues F310 and F457 in AdoIspS (Li et al., 2017). Through these analyses, it was further defined that the F310 residue is playing a more important role for isoprene emission compared with F457 and elucidated that isoprene synthase was likely derived from ocimene synthases through a process of active site reduction. Both AdoIspS F310 and F457, in fact, make van der Waals contacts with the DMADP substrate at the bottom of the hydrophobic cleft that constitutes the active site (Köksal et al., 2010) and are responsible for the reduction in the substrate‐binding pocket size that determines the specificity of IspS toward DMADP instead of geranyl diphosphate (GDP; Li et al., 2017). The homologue of AdoIspS F310 has also been demonstrated to play a pivotal role in the functional plasticity of monoterpene synthases in both angiosperms and gymnosperms, and it is part of a highly variable stretch of amino acids that are divergent among enzymes of this group (Gray et al., 2011; Kampranis et al., 2007). The second residue of the isoprene diagnostic tetrad, the homologue of AdoIspS S418, is also included in a highly variable region of monoterpene synthases. This region has been implicated in the formation of a characteristic kink in an alpha‐helix of *Salvia fruticosa* cineol synthase 1 involved in substrate selectivity and stereo‐specificity of terpene synthases (Kampranis et al., 2007; Köllner et al., 2004). While the first three residues of the isoprene diagnostic tetrad are clustering close to one another and participate to the definition of the substrate‐binding pocket of the enzyme, AdoIspS S477, the last amino acid of the H‐α1 loop, is located apart from the others at the top of the active site (Köksal et al., 2010).

Despite its discovery more than 60 years ago, the biological function of isoprene emission by plants is still not fully understood (Sharkey & Monson, 2017). Several studies with different approaches and plant species (Behnke et al., 2007; Sasaki et al., 2005; Velikova et al., 2011, 2016; Vickers et al., 2009; Xu et al., 2020) showed that isoprene emitters compared with nonemitters increased plant resistance to abiotic stresses such as heat, drought, and ozone treatments by maintaining photosystem II photochemistry stability and maintaining constant the stiffness of thylakoid membranes (Pollastri et al., 2019). Moreover, they decrease ROS content and lipid peroxidation (Ryan et al., 2014) and increase antioxidant levels (Vickers et al., 2009). In addition, recent studies by isoprene fumigation of Arabidopsis wild‐type plants revealed that isoprene functions as a signaling molecule increasing heat‐ and light‐stress responsive processes and inducing genes involved in the phenylpropanoid biosynthetic pathway (Harvey & Sharkey, 2016). Furthermore, the analysis of transcriptomic data of transgenic Arabidopsis plants overexpressing isoprene synthase in Arabidopsis found that isoprene synthase upregulates a set of genes involved in diverse metabolic pathways such as plant growth, heat, and drought stresses (Zuo et al., 2019).

The isoprene‐emitting species are scattered with little phylogenetic signal across the whole plant kingdom (Harley et al., 1999; Monson et al., 2013). Curiously, these isoprene‐emitting species are sporadically distributed also in the phylogenetic tree of angiosperm (http://www.es.lancs.ac.uk/cnhgroup/iso‐emissions.pdf; Sharkey et al., 2013), making it relatively difficult to identify IspS from primary sequence alone. The use of diagnostic amino acids is, therefore, a very convenient method to screen for putative *IspS* genes in public databases (Sharkey et al., 2013). So far, the majority of isoprene synthase genes have been isolated and characterized in dicots, mainly from Fabaceae. The integration of newly isolated isoprene synthase genes from a monocot, *A. donax* L., and from a dicot, *Casuarina equisetifolia* L., into the phylogenetic reconstruction further elucidated its monoterpene synthase origin and additionally uncovered the parallel evolution of isoprene synthases in angiosperms (Li et al., 2017; Oku et al., 2015).

The Arecaceae, commonly called “palm family,” is a plant family composed by around 2,600 monocotyledon species growing preferentially in tropical and subtropical regions worldwide (Baker & Dransfield, 2016). Some species have important economical values such as *Phoenix dactylifera* L., the date palm, whose fruits are mainly consumed in Arabic countries and distributed worldwide (Al‐Farsi & Lee, 2008); *Cocos nucifera* L., the coconut palm, is utilized to produce oil for hair and also for cooking; *Elaeis guineensis* Jacq., the oil palm, is planted in very large areas of tropical regions for the production of oil used for cooking and agroindustry and as lubricant in industrial applications (Barfod et al., 2015). The information currently available on isoprene emissions from Arecaceae stems mainly from general studies on volatile compounds dealing with specific areas, but no systematic survey of isoprene emissions of species from this family was carried out (Geron et al., 2006; Jardine et al., 2020). It has been reported that 20%–80% of the genera in the Arecaceae family emitted isoprene (Granier et al., 2004), but the emission capacities among different species can be very different. Given the high metabolic costs of isoprene emission (Zuo et al., 2019) and the impact that this hydrocarbon has on atmospheric chemistry (Guenther et al., 2006; Sindelarova et al., 2014), it would be highly desirable to select through screening of natural variation or genetic engineering, accessions of the most common palm species with reduced or absent isoprene emission. The lack of validated *IspS* genes from Arecaceae, however, hinders the possibility to resort to genetic approaches to this aim. Also, the identification of IspS from these family could provide highly active enzymes for isoprene production in bacterial systems or other heterologous systems (Chaves & Melis, 2018; Janke et al., 2020; Lv et al., 2016; Whited et al., 2010). So far, the functional importance of diagnostic tetrads was preferentially elucidated on two marker residues, F310 and F457 of AdoIspS, in *A. donax*, while only marginal evaluation of the other two tetrad residues (S418 and S477) was conducted (Li et al., 2017). Thus, elucidation of the IspS diagnostic tetrad in Arecaceae could provide novel insights into the function of the latter residues.

In the present study, we evaluated the isoprene emission capacity from 23 species representing 18 genera of the Arecaceae family. With the goal of screening for novel and potentially highly active enzymes for heterologous production of isoprene, several isoprene synthase genes were identified and functionally validated and phylogenetic reconstruction was carried out to determine the group of terpene synthases they belong to. Finally, the functional relevance of the novel diagnostic tetrad pattern identified for the first time in Arecaceae was further assessed by site‐specific mutagenesis in Arabidopsis, providing a novel model of the molecular mechanisms driving active site divergence between monocots and dicots and laying the foundation for sequencing‐driven screening of natural variation in IspS activity in Arecaceae.

## 2 MATERIALS AND METHODS

### 2.1 Plant materials and growing condition

In this study, different species (listed in Table S1) from the Arecaceae family growing in the Botanic Garden of Padova University (Italy) were analyzed. In addition, Arabidopsis Col‐0 wild‐type and transgenic plants generated from Col‐0 were used. Col‐0 and transgenic plants were grown in a growth chamber under standard long‐day condition (16‐hr light/8‐hr dark) at 23°C with light intensity of 100–120 µmol m^−2^ s^−1^ and 40% of relative humidity.

### 2.2 Genomic DNA isolation, total RNA extraction, and cDNA synthesis

Genomic DNA was isolated using 100 mg of fresh leaf material with the DNeasy Plant Mini Kit (Qiagen). The quality of extracted genomic DNA was assessed in 0.8% Agarose gel and quantified using Quant‐iT™ dsDNA Assay Kit (Thermo Fisher).

Total RNA was extracted from around 100 mg of frozen plant leaf material using the TRIzol reagent (Invitrogen) and treated with Amplification‐Grade DNase I (Sigma‐Aldrich^®^) for eliminating genomic DNA contamination. The integrity and quality analyses of extracted total RNA and cDNA synthesis using SuperScript™ III Reverse Transcriptase (Invitrogen™) were carried out as previous description (Poli et al., 2017).

### 2.3 Identification of novel isoprene synthases from Arecaceae family

Partial shotgun sequences of putative isoprene synthase from *Phoenix dactylifera* downloaded from NCBI were aligned with already available sequences from Li et al. (2017). Primers were designed to carry out genome walking using GenomeWalker™ Universal Kit from Clontech according to the manufacturer's instructions. Each cDNA synthesized as above was used as a template to amplify the full‐length coding region with Phusion High‐Fidelity DNA Polymerase (Thermo Fisher) and primer pairs designed based on the sequences obtained from genome walker method. Primers used here are listed in the Table S2. IspS sequences from *Bismarckia nobilis* Hildebrandt & H. Wendl., *Howea forsteriana* (F. Muell.) Becc., *Phoenix canariensis* H. Wildpret, *Sabal minor* (Jacq.) Pers., *Trachycarpus oreophilus* Gibbons & Spanner, and *Washingtonia filifera* (Rafarin) H. Wendl. have been deposited in GenBank with accession numbers MT512618–MT512623.

### 2.4 Phylogenetic reconstruction and tetrad comparison

The deduced amino acid sequences derived from conceptual translation of the cDNAs isolated from six Arecaceae species were aligned with the complete dataset of reviewed terpene synthases from the UniProt database. The protein sequences have been aligned with the Mafft server (Katoh et al., 2018), and the resulting multiple sequence alignment, containing 264 proteins (257 from UniProt, 6 from Arecaceae from this study and *A. donax* IspS; Li et al., 2017), has been trimmed with GBlocks (Talavera & Castresana, 2007) to remove poorly aligned regions using the following parameters: minimum number of sequences for a conserved position or a flanking position = 54, maximum number of contiguous nonconserved positions = 8, minimum length of a block = 5, allowed gap positions = with half, and use similarity matrices = yes. The best evolutionary model for the resulting alignment was selected with the SMS program (Lefort et al., 2017), and maximum likelihood reconstruction was conducted with the PhyML online server (Guindon et al., 2010), assessing the robustness of the inferred clades with approximate aBayes support. To analyze the occurrence of the tetrads in terpene synthases, all reviewed (257 proteins) and unreviewed (8,010 proteins) terpene synthases from the UniProt database were downloaded and only the proteins from angiosperms were aligned as described above. The alignments were manually annotated using the sequence from *Populus canescens*, and the columns corresponding to tetrad amino acids were manually extracted using the BioEdit program. Table 1 was compiled by retrieving the sequences of validated IspS enzymes from the accession numbers of the references listed in table. The proteins were aligned as described above and the tetrads deduced from the alignment based on their position in the sequence from *P. canescens*.

**TABLE 1:** Entire tetrad summary identified from different families in angiosperms

Tetrad	Family	Type	References
FVFT	Arecaceae	Monocotyledons	This study
FVFK	Casuarinaceae	Dicotyledons	Oku et al. (2015)
FVFN	Convolvulaceae, Elaeocarpaceae, Fagaceae	Dicotyledons	Ilmén et al. (2015)
FSFN	Fabaceae, Moraceae, Myrtaceae, Salicaceae	Dicotyledons	Oku et al. (2015), Sharkey et al. (2013)
FSFS	Poaceae, Anacardiaceae	Dicotyledons, monocotyledons	Ilmén et al. (2015), Li et al. (2017)

### 2.5 Plasmid construction, plant transformation, and positive selection

The full‐length cDNAs of *HfoIspS*, *PcaIspS,* and *SmiIspS* were amplified with Phusion High‐Fidelity DNA Polymerase (Thermo Fisher) and corresponding primer pairs, and individually cloned into Gateway vector pENTR/D‐TOPO (Invitrogen) (pENTR_HfoIspS, pENTR_PcaIspS, and pENTR_SmiIspS).

Two of the residues of the “IspS diagnostic tetrad” indicated by Sharkey (Sharkey et al., 2013); F338, S446, F485, and N505 of poplar IspS), V420 and T479 in PcaIspS of *P. canariensis*, were subjected to site‐directed mutagenesis using the QuikChange Site‐Directed Mutagenesis Kit (Stratagene) and the wild‐type clone pEN_PcanIspS as a template. In total, four single mutations (V420S, T479K, T479N, and T479S) and two double mutations (V420S+T479S and V420S+T479N) were performed.

A total of nine pENTR clones including the six IspS diagnostic tetrad mutations, the wild‐type pENTR_PcaIspS from *P. canariensis* (PcaIspS‐WT), and the two CDS from *Sabal minor* and *Howea forsteriana* (pENTR_SmiIspS and pENTR_HfoIspS) were recombined into the destination vector pK7WG2 (Karimi et al., 2002) through LR reaction using LR clonase II (Invitrogen). These final constructs were transformed individually into *Agrobacterium tumefaciens* strain GV3101‐pMP90RK by electroporation and then further transformed into *Arabidopsis thaliana* Col‐0 ecotype using the floral dip method (Clough & Bent, 1998). The positive transformants were screened by sowing sterilized seeds on solid MS (Murashige and Skoog) medium containing 50 mg/L of kanamycin, and the presence of transgenes was further confirmed by PCR using the genomic DNA extracted individually with the CTAB method (Doyle & Doyle, 1987). The PCR amplification conditions were as follows: 95°C for 2 min, 35 cycles at 94°C for 40 s, 60°C for 30 s, and 72°C for 2 min. The primers used for plasmid constructions and PCR amplifications are listed in Table S2.

### 2.6 Proton transfer reaction mass spectrometry (PTR‐MS) measurements

The volatile compound emission for each species of Arecaceae family collected (see Table S1), Col‐0 and transgenic Arabidopsis lines, was measured with a commercial PTR‐ToF 8000 apparatus from IONICON Analytik GmbH. The whole procedure from sample preparation and data acquisition was performed as former description (Li et al., 2020). Briefly, leaf parts were incubated in a sealed 20‐ml vial for 3 hr. Then, the concentration of the headspace of detached leaves parts was measured in the dark to prevent further isoprene emission. The concentration of the isoprene present in the headspace was normalized based on the dry weight of the leaf part used for the measurements and calculated on an hourly base by dividing for the total number of collection hours (3).

### 2.7 Structure modeling

Structural modeling was carried out with the Swiss‐model server (Arnold et al., 2006) using as model the crystal structure of *P. canescens* IspS complexed with magnesium ions and the substrate analogue dimethylallyl‐*S*‐thiolodiphosphate as template (pdb accession: 3n0g, Köksal et al., 2010). In addition to the WT *P. canariensis* IspS, also 3D structure of the single mutants T479K, T479N, T479S, and V420S was modeled. Hydrogen bonds were inferred using the FindHBonds tool of Chimera v.1.14 (Pettersen et al., 2004) using H‐bonds constraints relaxed by 0.4 Å and 20°.

### 2.8 Statistical analyses

Unless otherwise stated, for each statistical test an *α* = .05 was applied to determine statistical significance. For statistical analysis of differences in total isoprene emission comparison between each Arecaceae species and the negative control (nonemitter Arabidopsis Col‐0), the one‐tail Student *t* test with the Benjamini–Hochberg correction for multiple testing was used. For statistical analysis of differences in total isoprene emission comparison between each mutation of the PcaIspS protein and the WT control (PcaIspS‐WT), the one‐tail Student *t* test was used. All analyses were run in R.

## 3 RESULTS

### 3.1 Isoprene emission is a widespread trait in the Arecaceae family

**FIGURE 1:**
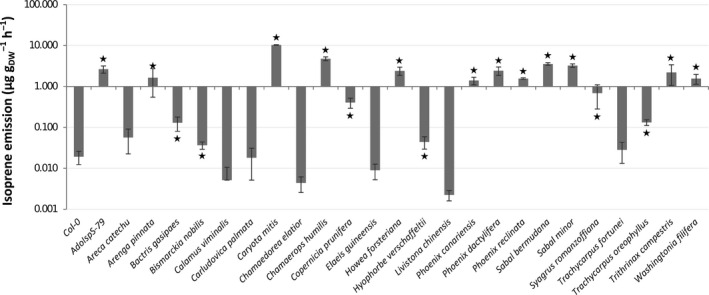
Isoprene emission level shown in logarithmic scale among different species in the Arecaceae family. Arabidopsis Col‐0 is shown as negative control (low emitter), the AdoIspS‐79 transgenic Arabidopsis line as positive control for isoprene emission and *Carludovica palmata* selected as an outgroup for the Arecaceae family. Stars on top of bars indicate statistically significant different means (higher than Arabidopsis Col‐0) based on Student *t* test (*p* < .05) followed by a Benjamini–Hochberg false discovery rate correction (*p* < .05)

PTR‐Tof‐MS was applied to measure isoprene emission from fresh leaves of 23 Arecaceae species, including Arabidopsis Col‐0 as a negative control (low emitter) and the AdoIspS‐79 transgenic Arabidopsis line as a positive control (Li et al., 2017). The majority of species emitted higher amounts of isoprene relative to the positive control AdoIspS‐79 (Figure 1). To evaluate which species emitted isoprene based on the concentration of the gas in the headspace of the sample, a Student *t* test with the Benjamini–Hochberg false discovery rate correction was used (Table S1), indicating that all species from Arecaceae family examined here except six (*Areca catechu* L., *Calamus viminalis* Willd., *Chamaedorea elatior* Mart., *E. guineensis*, *Livistona chinensis* (Jacq.) R.Br., and *Trachycarpus fortunei* (Hook.) H. Wendl.) were able to emit significant amounts of isoprene in the range from *Bismarckia nobilis* (3.7 × 10^−2^ µg g_DW_^−1^ hr^−1^) to *Caryota mitis* Lour. (10.13 µg g_DW_^−1^ hr^−1^). *Carludovica palmata* Ruiz & Pav., used here as outgroup, also emitted low amounts of isoprene, not significantly different from those emitted by *A. thaliana* Col‐0.

### 3.2 Isolation and functional validation in *Arabidopsis* of novel isoprene synthase genes from Arecaceae

**FIGURE 2:**
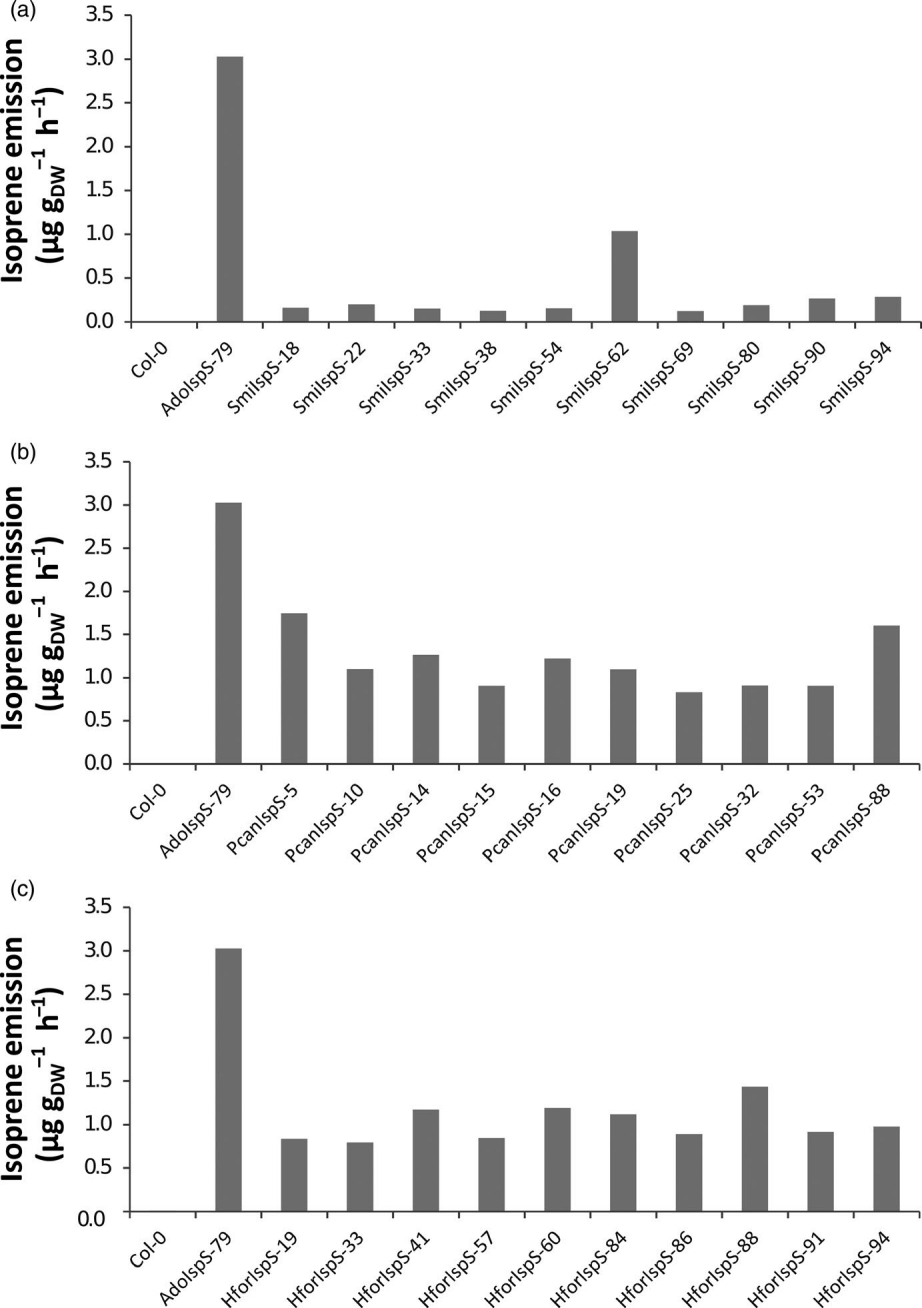
Isoprene emission (single measurement screening) of the top ten T1 transgenic Arabidopsis lines for three selected transgenes. (a) Transformants overexpressing *SmiIspS*, (b) transformants overexpressing *PcaIspS*, and (c) transformants overexpressing *HfoIspS*. Col‐0 is used as negative emitter and transgenic Arabidopsis AdoIspS from *Arundo donax* as positive control (constitutive emitter)

Six independent isoprene synthase genes were isolated from the following species, chosen to encompass six different genera and two tribes in the family: *Bismarckia nobilis*, *Howea forsteriana*, *Phoenix canariensis*, *Sabal minor*, *Trachycarpus oreophilus,* and *Washingtonia filifera*, and through conceptual translation, all sequences were deduced to code for 585‐amino‐acid‐long proteins. The sequence alignment indicated that all these putative isoprene synthases shared the same novel diagnostic tetrads as 312F, 420V, 459F, and 479T. The proteins share 89%–98% amino acid sequence identity among them, and sharing around 53% with AdoIspS from *A. donax* and 49% with *Pueraria montana* (Lour.) Merr. (Figure S1). The first 21 amino acids of all these isoprene synthases were predicted as the transit peptides for chloroplast import using the program ChloroP 1.1 (Emanuelsson et al., 1999). The full‐length cDNA of isoprene synthase genes from *H. forsteriana*, *P. canariensis,* and *S. minor* were independently overexpressed under the CaMV 35S promoter in Arabidopsis Col‐0. The presence of the isoprene synthase transgenes was verified by PCR, and isoprene emissions from 90 independent transgenic lines for each isoprene synthase from different species were measured using PTR‐Tof‐MS, and the emission levels from the top 10 lines are shown in Figure 2. Indeed, even though the emission levels of different lines corresponding to isoprene molecular mass 69.069 from all the transformants were very different due to positional effects (Li et al., 2020), statistical analyses clearly showed that all these transgenic lines emitted isoprene compared with Col‐0 wild‐type plants (Student *t* test *p*‐value < .0001). Thus, these genes are indeed isoprene synthase genes identified for the first time from the Arecaceae family.

### 3.3 Arecaceae IspSs belong to TPS‐b clade 2 terpene synthases and are characterized by a novel diagnostic tetrad

**FIGURE 3:**
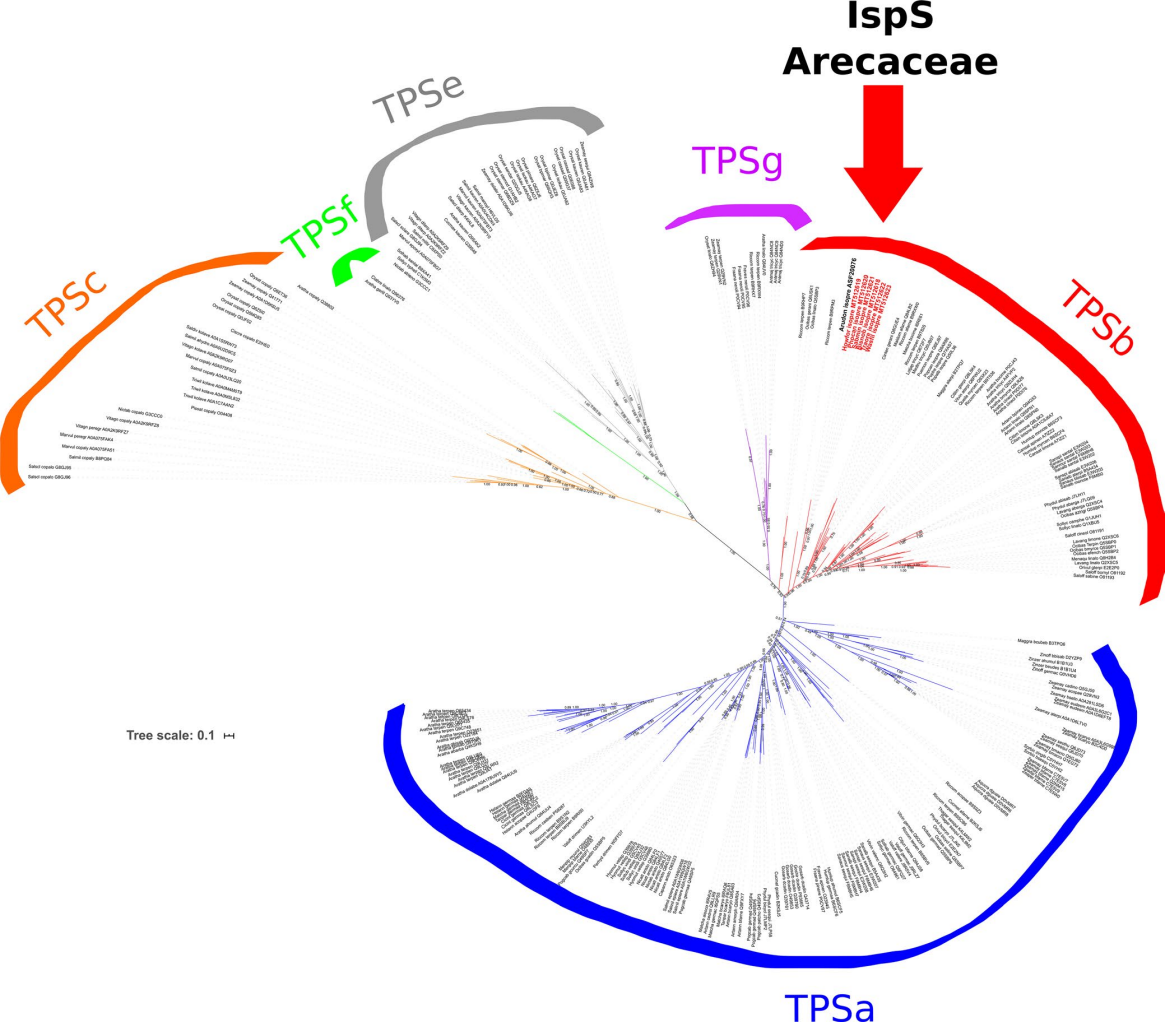
Phylogenetic reconstruction using maximum likelihood for selected angiosperm terpene synthase proteins from classes a, b, c, e, f, and g. In TPS‐b clade 2, the names in bold red indicate the Arecaceae IspSs, while the IspS protein from *Arundo donax* is in bold black. Numbers close to the branches are approximate Bayes support values. The abbreviations used and the complete list of taxa is available in Table S3

Based on the available sequences from all major angiosperm terpene synthases, phylogenetic reconstruction of newly identified isoprene synthases from Arecaceae family using maximum likelihood was carried out and concluded that they belong to TPS‐b clade 2 terpene synthases. Furthermore, all of the novel sequences were grouped together and formed a monophyletic group with AdoIspS from *A. donax*, another monocot species (Figure 3). To define the evolution of this important diagnostic tetrad along angiosperms, the summary of different families in relation with these tetrads was constructed (Table 1). It indicated that there are overall two kinds of tetrads present in monocots and four kinds in dicots, and one kind (FSFS) is present in both clades. The tetrad in Arecaceae represented a novel pattern, which was never identified before. In addition, this new integration in monocot sequences further indicated that the amino acids of the tetrad in position 2 could be either S (serine) or V (valine) in both monocots and dicots. However, the forth position of the tetrad could be either S (serine) or T (threonine) in monocots, and K (lysine) or N (asparagine) or S (serine) in dicots. Thus, the forth position of the tetrad has higher flexibility as it can accommodate in the diagnostic tetrad more types of amino acidic residues than the second position of the tetrad. To check the distribution of the diagnostic tetrads across angiosperms, both reviewed and unreviewed TPS proteins from UniProt were analyzed. According to the annotations of the 257 reviewed proteins, the FVFT tetrad is present only in the newly identified IspS from Arecaceae (Table S3). Also extending the search to the about 8,000 unreviewed dataset of TPS synthases in UniProt, the FVFT tetrad resulted specific to Arecaceae IspS only. Interestingly, also the FSFN tetrad appears to be still specific to IspS, with only a few uncharacterized sequences from Fabaceae and Myrtaceae that are presumably bona fide novel isoprene synthases given the perfect match between tetrad and family they belong to Table S3.

### 3.4 Functional specificity of diagnostic tetrad residues of isoprene synthase from *P. canariensis* in vivo

**FIGURE 4:**
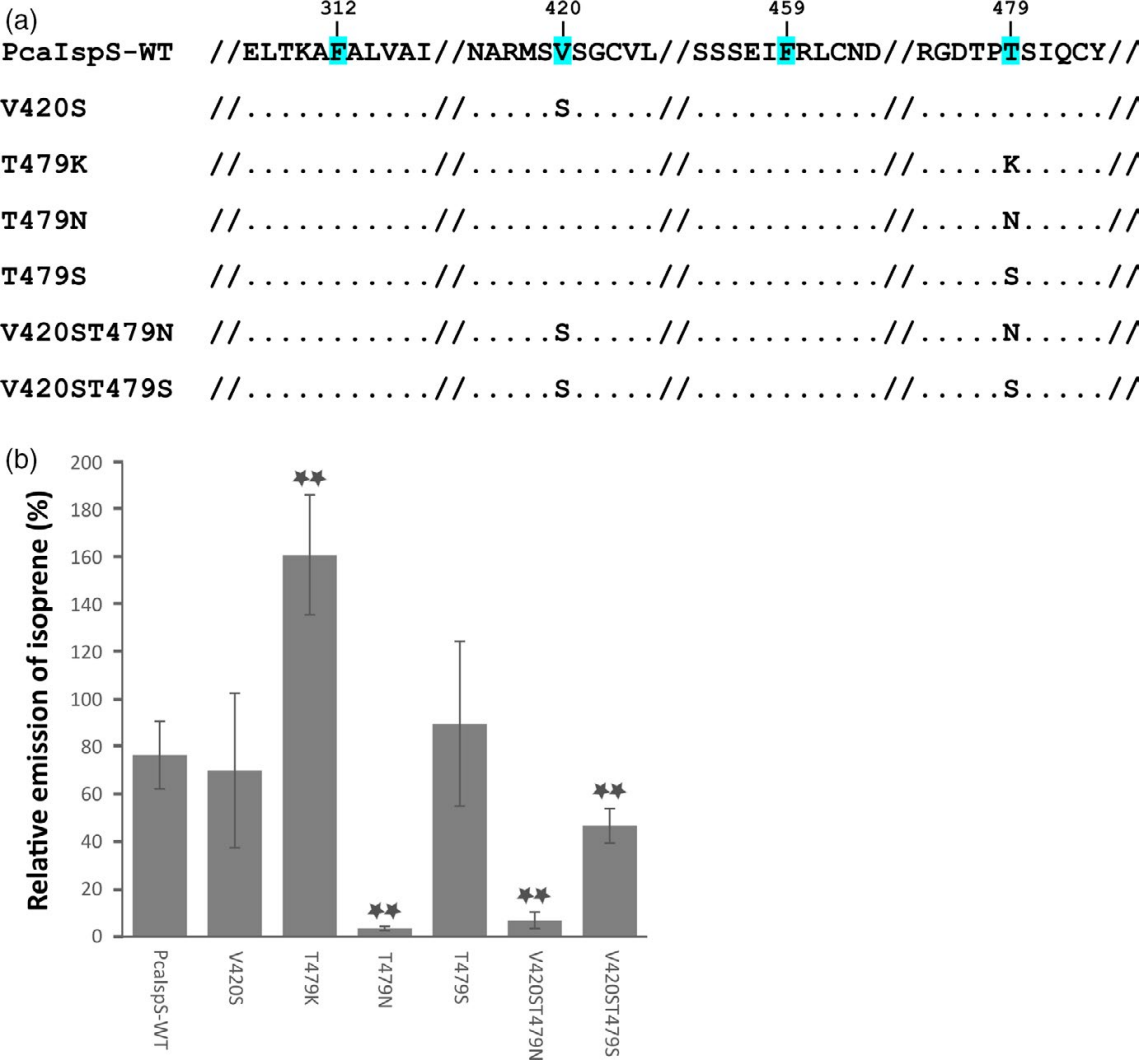
Scheme of diagnostic tetrad mutations in PcaIspS protein and relative isoprene emission of transgenic Arabidopsis lines transformed with wild‐type *PcaIspS* and mutations in diagnostic residues V420 and T479. (a) The four amino acids with blue background are those of the diagnostic tetrad. The numbers shown above the tetrad residues correspond to the positions in the wild‐type PcaIspS, the dots indicate amino acids that are identical to that of wild‐type PcaIspS, and the symbol “//” marks parts of amino acid sequences not shown due to space limitation. (b) Relative isoprene emission normalized with the highest emitting line in transgenic Arabidopsis PcaIspS‐WT plants. The top six lines for each transformation were used for this analysis. Stars on top of bars indicate significant different means (*p* < .01)

Site‐directed mutagenesis was performed for PcaIspS to change either S418 or S477 or both together to already existing diagnostic tetrads identified until now in isoprene synthases in the corresponding position. Thus, the following six diagnostic tetrad patterns through either single amino acid mutation (V420S, T479N, T479K, and T479S) or double mutations (V420ST479N and V420ST479S) in position V420 and T479 of wild‐type PcaIspS were generated, and subsequently, these constructs were individually transformed into Arabidopsis Col‐0 wild‐type plants and volatile compound emissions of at least 80 transgenic lines for each transformation independently were evaluated. In the case of transgenic plants carrying either T479N or V420ST479N mutation, isoprene emission was very low with about 5% or 9% emission relative to wild‐type PcaIspS, but the statistical analyses clearly indicated that the emission levels were significantly higher than that of Col‐0 (*p*‐value: 2.827 × 10^−5^ for T479N and .0023397 for V420ST479N). Isoprene emission level for V420ST479S mutation was also significantly lower with about 61% relative emission to that of wild‐type PcaIspS (*p*‐value: 6.416 × 10^−8^). Similar emission levels to wild‐type PcaIsps were detected for T479S or V420S mutation (*p*‐value > .01). Interestingly, a significantly higher emission was detected for the T479K mutation (*p*‐value: 6.037 × 10^−8^) (Figure 4). Thus, these analyses implied that whenever T (threonine) at position 479 was changed to N (asparagine) irrespective of V (valine) or S (serine) at position 420, the mutations caused a strong decrease in isoprene emission. However, in V420S or T479S mutations, isoprene emission was more or less maintained as that of wild‐type PcaIspS, and isoprene emission was significantly lower than that of wild‐type PcaIspS in T479N or V420ST479N or V420ST479S mutation.

### 3.5 Variation of hydrogen bonding among enzyme variants at tetrad sites

**FIGURE 5:**
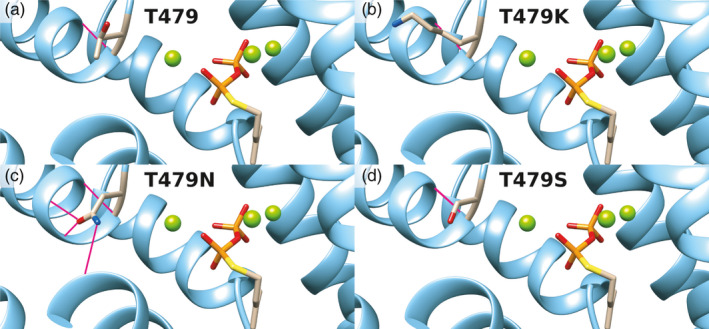
Structural modeling of *Phoenix canariensis* IspS enzyme based on the crystal structure of *Populus canescens* (pdb accession: 3n0g). The images depict the ribbon representation of the upper part of the active site of PcaIspS‐WT (a), and of mutants T479K (b), T479N (c), and T479S (d). The three magnesium atoms present in the active site are displayed as green spheres. The side chains of amino acids in position 479 (top‐left side of the images) and the substrate analogue dimethylallyl‐*S*‐thiolodiphosphate (right side of the images, among magnesium atoms) are represented as sticks. Carbon atoms are in sand color, sulfur in yellow, phosphorus in orange, oxygen in red, and nitrogen in blue. Hydrogen bonds are represented as pink lines connecting donor and acceptor atoms. Images were drawn with the UCSF Chimera v.1.14 software

The 3D structures of *P. canariensis* WT IspS and of the single mutants T479K, T479N, and T479S modeled on the crystal structure of *P. canescens* had low values of the QMEAN *Z*‐scores (QMEAN < −2.42), indicating good agreement between the model and the experimental structure and suggesting that the structural changes leading to the different activities are local. Analysis of the H‐bond patterns in either WT or mutant enzymes indeed identified changes in the number and position of the H‐bonds done by the residue in position 479 among enzymes (Table S4). The H‐bonds of the residues in position 479 and cysteine 483 were present in the WT and all the mutants in the 479 series, suggesting they were not the cause for the differences among enzyme activities. In addition, the bond between threonine 477 and position 479 could not account for the differences among mutant enzymes, as it was present in all the mutants but not in WT. The H‐bonds differing among enzymes were instead mainly those connecting the helixes H1α, H, and I. In mutant T479N, the mutant asparagine residue anchored directly the beginning of the H1α helix (I481) to the H helix (D464) and no direct link was observed between D464 and any residue of the I helix (Figure 5, Table S4). By contrast, the mutant T479K side chain was tucked away deeper between the H1α helix and the H helix, allowing the formation of a three‐way link among helixes H1α, H, and I: The H and I helixes were connected through the S468‐E492 H‐bond, while H and H1α helixes were connected through a flexible double bond S468‐K479 and K479‐N482 where the NH_4_^+^ residue of the K479 side chain acts as a bridge. Also in the WT and T479S mutant, the H and I helixes were connected by an H‐bond involving S468, but this time with the K496 residue, one turn higher in the I helix (Figure 5, Table S4).

## 4 DISCUSSION

In total, 17 species from Arecaceae out of the 23 examined in this study have the capacity to emit more isoprene than that of Arabidopsis Col‐0, and among them, *Caryota mitis* is the highest emitter. These results support and extend the former estimates that 20%–80% of species in this family are emitting isoprene (Granier et al., 2004) and further confirm species in the *Caryota* genus as strong isoprene emitters among woody plants (Klinger et al., 2002). The fact that in our study a relatively high proportion of the species (74%) emits isoprene might be in part derived from technical differences among studies. In our analyses, we applied state of the art analytical methods based on PTR‐Tof‐MS, which, with its high sensitivity, has the capacity to detect as little as low parts per trillion by volume (ppt_v_) amounts of volatile compounds (Jordan et al., 2009). It is, therefore, possible that former estimations of the percentage of isoprene‐emitting Arecaceae might have been underestimated because of the use of different methods such as cartridge plus gas chromatography with flame ionization detector (GC‐FID) (HP3390) (Harley et al., 2004).

Our estimations of isoprene emission are comparable with those of previous studies summarized in the database of the Lancaster Environment Centre (http://www.es.lancs.ac.uk/cnhgroup/iso‐emissions.pdf and references therein), although in the two cases where the same species were sampled, the emissions we measured are lower than previously reported. In the case of *Washingtonia filifera*, for instance, our results indicate an average isoprene emission of 1.6 ± 0.4 µg g_DW_^−1^ hr^−1^. This figure is about 10 times less than the average emission reported for the same species in the Lancaster database (9.9–11 ± 12 µg g_DW_^−1^ hr^−1^), but in this case the high error on the measurements makes the results difficult to compare. In the case of *Phoenix dactylifera*, the date palm, our results were about six times lower than previously reported (2.4 ± 0.6 µg g_DW_^−1^ hr^−1^ vs. 15 ± 1 µg g_DW_^−1^ hr^−1^), possibly a result of different growth conditions among the analyzed samples.

Large variations in the ability to emit isoprene are not surprising among related species, as previous work indicated that even within the *Populus* genus, different species grown in a common garden have varying levels of isoprene emission (Ahrar et al., 2015; Schnitzler et al., 2010). The survey in the Arecaceae family we carried out did not evidence large variations in average isoprene emission among species of the *Phoenix* genus, but the limited species sampling linked to our experimental design does not allow reaching general conclusions. Among genera, however, the differences were very large. The two most important species from a commercial point of view, for instance, the date palm (*P. dactylifera*) and the oil palm (*E. guineensis*), differed by about two orders of magnitude in emission. Despite being very low (not significantly different from those of Arabidopsis Col‐0), the isoprene emission levels of *E. guineensis*, however, can be still relevant at the global level, as this species alone covers an area of more than 16 million hectares, corresponding to about 10% of the world's permanent cropland (Pirker et al., 2016). The impact of even relatively small variations among cultivars of this species in isoprene emission can thus be extremely large. To date, however, the lack of characterized IspS genes from Arecaceae hindered the inclusion of isoprene emission among the traits object of genetic improvement.

In line with the ability to emit isoprene, the six putative *IspS* genes newly identified in Arecaceae share the basic features of isoprene synthases: two marker residues of phenylalanines in the active pocket site (F312 and F459; Sharkey et al., 2013) and heavy‐metal‐binding motifs (DDXXD and DTE/NSE; Köksal et al., 2010). Surprisingly, however, they display also a novel combination of diagnostic tetrad residues, FVFT, which has not been described in any other family until now (Li et al., 2017; Sharkey et al., 2013). The fact that the isolated genes code for enzymes having exclusive isoprene synthase activity is demonstrated by the fact that the transgenic Arabidopsis plants overexpressing three of these putative isoprene synthase genes are able to emit only isoprene. This implies that they are functional orthologs of characterized isoprene synthase genes from *Populus alba* L., *P. nigra* L., *C. equisetifolia*, and *Metrosideros polymorpha* J.R. Forst. ex Hook. f. (Fortunati et al., 2008; Oku et al., 2015; Sasaki et al., 2005; Yeom et al., 2018). The conservation of the FVFT tetrad in all six *IspS* genes isolated, thus, confirms its diagnostic value at the family level (Li et al., 2017), as the species they are derived from encompass six different genera and two tribes in the family.

It has been previously reported that the amino acids in positions 2 and especially 4 of the tetrad are more variable than those in positions 1 and 3, which are nearly exclusively phenylalanine residues. However, positions 2 and 4 are also functionally relevant, as their substitution strongly impairs or abolishes isoprene emission (Li et al., 2017). So far, four different kinds of diagnostic tetrads have been identified from one Poaceae family in monocots and 10 families in dicots (FSFS, FSFN, FVFN, and FVFK, Ilmén et al., 2015; Miller et al., 2001; Oku et al., 2015), which are different from the current identification from Arecaceae family (FVFT) and suggest the existence of functional constraints in all positions of the tetrad.

Keeping in mind the limited composition of amino acids in different positions of the diagnostic tetrads and the recurrence of some of the same tetrads in different families (even between monocot and dicot such as FSFS), we tested whether the novel tetrad from PcaIspS can be functionally substituted by other families' tetrads or hybrid tetrads not identified in nature. The comparison of different emissions is intrinsically complex in a heterologous system like *A. thaliana*, where differences in transgene copy number and expression levels are factors that can make interpretation of the results difficult. Ideally, our assay in *A. thaliana* would need independent confirmation also with in vitro assays with purified recombinant proteins, but, unfortunately, our attempts to express three of the Arecaceae IspS in *Escherichia coli* failed due to lack of expression (data not shown). The use of alternative expression systems (e.g., in planta recombinant protein expression, Lindbo, 2007; Rademacher et al., 2019) could circumvent this problem, but it is time‐consuming. Thus, to mitigate as much as possible the uncertainties with the assay in *A. thaliana*, we analyzed a high number of transgenic lines obtained with a strong promoter, two measures which should contribute to limiting the confounding effects of transgene copy number and integration site. Additionally, we took a comparative approach, which minimizes the possible incidence of factors not related to the variability among transgenic lines like codon usage as well as translation and/or post‐translational modifications. Thus, although caution must be used in the interpretation of the results, the comparison among transgenic lines provides some interesting indications on the differences among IspS mutants and the respective WT gene copy. In general, PcaIspS appeared quite tolerant toward substitutions, as the majority of the exchanges preserved significant levels of isoprene emission compared with WT. Only the combination of FSFN or FVFN in PcaIspS almost abolished isoprene emission in the *A. thaliana* assay, analogously to what happened for the FSFN mutation in AdoIspS of *A. donax*, another monocot species (Li et al., 2017). Even though these two combinations (FSFN and FVFN) are natively present at least in three different families of dicots (Ilmén et al., 2015; Sharkey et al., 2005), these combinations of tetrads do not seem to function in monocot species, suggesting that they might be dicot‐specific combinations. Taken together, these results point to the presence of a complex pattern of interactions among IspS residues besides those of the diagnostic tetrad. The results of the site‐directed mutagenesis in conjunction with the structural modeling, in particular, suggest that the fourth position of the tetrad seems to have a significant effect on enzyme activity, in line with the pivotal roles of its homologue in the specification of TPS activity (Kampranis et al., 2007). Based on these results, the variation of the number and type of hydrogen bonds formed by the side chain of the residue at this position could affect the interconnections among helices H1α, H, and I.

We propose that substitutions like T479N, abolishing the hydrogen bonds between position 479 and residues of the I helix and/or establishing new bonds with helix H, can decrease the activity of the enzyme by hindering the conformational changes associated with the open–closed transition of IspS. This is consistent with the capping function of helix H1α for IspS active site and its proposed role in diphosphate positioning/sensing during transition to the closed conformation (Kampranis et al., 2007; Li et al., 2017). On the other hand, the increased enzyme activity observed in the T479K mutant in our Arabidopsis in vivo assay is compatible with the higher flexibility afforded by the longer side chain of lysine in its bridging function between helixes H1α and H and the conservation of the connection between helixes H and I through residue S468. The constraints for the second position of the tetrad are possibly more relaxed, but they have been tested here only for a pretty conservative substitution (i.e., V420S). As in TPS the homologue of the 420 residue interacts with the substrate analogue DMASPP through its carbonyl oxygen atom (Köksal et al., 2010), one can expect this position to be tolerant to mutations. As the variable region of TPS this amino acid belongs to has been implicated in the formation of a functionally relevant kink in TPS helixes (Kampranis et al., 2007), it will be interesting to further test by saturating mutagenesis whether the helix propensity of residues at this position is relevant for IspS activity.

Interestingly, the two new combinations of mutations (FVFS and FSFT), not identified in the plant kingdom yet, have the capacity to emit similar amount of isoprene as that of wild‐type PcaIspS based on our in vivo assay. This observation, together with the constance of the tetrads at the family level, suggests that the whole functional space of mutations compatible with IspS activity has not been fully sampled in nature and that evolution of the enzyme may be at least in part canalyzed by the particular set of amino acidic substitutions that stochastically took place in the past. Interestingly, the FVFK mutation in PcaIspS, naturally present in IspS of Casuarinaceae family in dicot (Oku et al., 2015), seems to dramatically increase isoprene emission compared with wild‐type PcaIspS. As isoprene biosynthesis is metabolically expensive (Sharkey & Yeh, 2001), this variation in enzymatic activity could reflect the existence of selective constraints on the levels of isoprene emission to prevent excessive carbon and energy loss. Indeed, the activities of IspS enzymes from different families show a relatively large range of variation and low affinity of the enzymes for their substrate (Li et al., 2019; Silver & Fall, 1995; Yeom et al., 2018), indicating that adaptive trade‐offs may prevent the attainment of maximal potential activity. This observation has important implications from an applicative point of view, as it indicates that there is room to select enzymatic variants with enhanced isoprene emission capability. As the recent advancements of the engineering approaches aimed at the production of isoprene in yeast and especially *E. coli* mainly focused on the steps upstream of IspS (Kim et al., 2016; Wang et al., 2017), the results obtained in this work may help in the identification of novel isoprene synthase genes with improved catalytic activity. On the other hand, isoprene emission has also large impacts on air quality, and atmospheric chemistry at the global level (Guenther et al., 2006; Sindelarova et al., 2014). Recently, hybrid poplar engineered to prevent isoprene emission has been demonstrated to have normal plantation biomass production, thus indicating that isoprene emission can be suppressed without significant drawbacks for productivity, but with substantial advantages for air quality (Monson et al., 2020). The identification of isoprene synthases from Arecaceae opens the way to analogously reduce/suppress isoprene emissions from *E. guineensis* and other widely cultivated palm species, thus ensuring a higher environmental sustainability of these important crops. This could be directly achieved by screening of natural variation for IspS gene expression in the genetic pool of the species. Alternatively, given the availability of protocols for genetic transformation of *E. guineensis*, genome editing could be applied (Yarra et al., 2019).

In conclusion, in this study we assessed the various isoprene emission levels among different species in Arecaceae, an economically important family of monocotyledonous plants. Importantly, the isolation of six novel isoprene synthases and the identification of the brand‐new diagnostic tetrad pattern further broadened our knowledge on isoprene synthases in plants and pave the way to the selection of new varieties with reduced emission and improved environmental friendliness. Furthermore, in vivo analyses of different combinations of amino acids in diagnostic tetrad using currently available information from different families demonstrated that the novel combinations of diagnostic tetrads are functional and could be present in unidentified species in nature. In a particular combination, the isoprene emission could even be increased in the majority of the transgenic lines tested, suggesting a rationale for targeted improvement of IspS activity for metabolic engineering.

## CONFLICT OF INTEREST

None declared.

## AUTHOR CONTRIBUTIONS

ML and CV conceived and designed the work. ML and JX carried out most of the experimental work with the help of FL for transgenic plants, IK for VOC measurement, and MV for Arecaceae species sampling. ML and CV wrote the manuscript. JX, FL, IK, FB, MV, and BB corrected, read, and proofed the manuscript.

## Supporting information

Supplementary Material

Table S3

Table S4

## Data Availability

The data that support the findings of this study are openly available in TreeBASE at http://purl.org/phylo/treebase/phylows/study/TB2:S27264, reference number TB2:S27264.
