# Supplementary material for: Evolution of isoprene emission in Arecaceae (palms)
Source: Evol Appl. 2020 Dec 14;14(4):902–14. doi: 10.1111/eva.13169 (PMC8061277; doi:10.1111/eva.13169)

**Functional relevance of diagnostic tetrad composition for isoprene emission: a case study in Arecaceae species**

**Summary of supplementary tables and figures:**

Table S1. Average isoprene emission of different Arecaceae species and controls (Col-0: negative emitter and AdoIspS-79: positive emitter).

| **Species name** | **Normalized isoprene emission (µg g_DW_^-1^ h^-1^)** | **Stdev** | **P-value^a^** | **Corrected P-value^b^** |
| --- | --- | --- | --- | --- |
| *Col-0* | 0.0192 | 0.0068 |  |  |
| *AdoIspS-79* | 2.6403 | 0.5300 | 0.000510278 | 0.001895318 |
| *Areca catechu* L. | 0.0568 | 0.0342 | 0.067574149 | 0.07638817 |
| *Arenga pinnata* (Wurmb) Merr. | 1.6425 | 1.0924 | 0.030866656 | 0.03821586 |
| *Bactris gasipaes* Kunth | 0.1301 | 0.0499 | 0.009451828 | 0.01755339 |
| *Bismarckia nobilis* Hildebrandt & H.Wendl. | 0.0366 | 0.0075 | 0.020236983 | 0.03095068 |
| *Calamus viminalis* Willd. | 0.0051 | 0.0055 | 0.025275949 | 0.03458814 |
| *Carludovica palmata* Ruiz & Pav. | 0.0181 | 0.0130 | 0.452912563 | 0.4710291 |
| *Caryota mitis* Lour. | 10.3135 | 0.1584 | 1.87592E-08 | 4.88E-07 |
| *Chamaedorea elatior* Mart. | 0.0044 | 0.0018 | 0.011217967 | 0.01944448 |
| *Chamaerops humilis* L. | 4.7914 | 0.5143 | 4.38576E-05 | 0.00022806 |
| *Copernicia prunifera* (Mill.) H.E.Moore | 0.4069 | 0.1132 | 0.002039104 | 0.004418059 |
| *Elaeis guineensis* Jacq. | 0.0090 | 0.0037 | 0.042926614 | 0.05073145 |
| *Howea forsteriana* (F.Muell.) Becc. | 2.4231 | 0.5522 | 0.000828811 | 0.002251426 |
| *Hyophorbe verschaffeltii* H.Wendl*.* | 0.0443 | 0.0147 | 0.027530826 | 0.03579007 |
| *Livistona chinensis* (Jacq.) R.Br. | 0.0022 | 0.0006 | 0.006477531 | 0.01295506 |
| *Phoenix canariensis* H.Wildpret | 1.3953 | 0.2921 | 0.000614285 | 0.001996426 |
| *Phoenix dactylifera* L. | 2.4262 | 0.5594 | 0.000865933 | 0.002251426 |
| *Phoenix reclinata* Jacq. | 1.5529 | 0.0746 | 1.8826E-06 | 2.45E-05 |
| *Sabal bermudana* L.H.Bailey | 3.5649 | 0.2579 | 9.23266E-06 | 8.00E-05 |
| *Sabal minor* (Jacq.) Pers. | 3.2526 | 0.3053 | 2.59959E-05 | 0.000168973 |
| *Syagrus romanzoffiana* (Cham.) Glassman | 0.6895 | 0.4084 | 0.023375976 | 0.0337653 |
| *Trachycarpus fortunei* (Hook.) H.Wendl. | 0.0282 | 0.0150 | 0.197484022 | 0.213941 |
| *Trachycarpus oreophilus* Gibbons & Spanner | 0.1327 | 0.0206 | 0.000412053 | 0.001785563 |
| *Trithrinax campestris* (Burmeist.) Drude & Griseb. | 2.2236 | 1.1629 | 0.015202124 | 0.02470345 |
| *Washingtonia filifera* (Rafarin) H.Wendl. | 1.5561 | 0.4214 | 0.001606166 | 0.003796392 |

^a^: Student t-test, P < 0.05

^b^: Benjamini-Hochberg false discovery rate, P < 0.05

**Table S2**. Primers used for this study.

| **primer name** | **sequence (5' - 3')** | **purpose** |
| --- | --- | --- |
| EguIspS_GW-Rev1 | GGAAACCCGTGCTCTCTAAGGAGTC | GenomeWalker |
| EguIspS_GW-Rev2 | GTCATCTTTGAACATCAAGTCTGCCCTC | GenomeWalker |
| IspS_For | CCAAACAATAGAAGTAGCTCAAGC | full-length cDNA cloning |
| IspS_Rev | CCCTTCTACACATTGCTGACC | full-length cDNA cloning |
| PcaIspS_For | CACCAAACAATAGAAGTAGCTCAAGC | pENTR cloning |
| PcaIspS_Rev | CTACACATTGCTGACCACAAACC | pENTR cloning |
| SmiIspS_For | CACCATGGCACTCTCTACATGCTTCGCA | pENTR cloning |
| SmiIspS_Rev | CTACACATTGCTGACCACAAACCT | pENTR cloning |
| HfoIspS_For | CACCATGGCATTGTCTACATGCTGTGCA | pENTR cloning |
| HfoIspS_Rev | CTACACATTGCTGACCACAAGCCC | pENTR cloning |
| pK7WG2_For | GACAATCTGATCCAAGCTCAAGC | positive screening |
| V420S_For | ATCTAAGCAATGCCCGGATGTCATCTTCAGGCTGCGTCCTATTAGTCCATGC | site-directed mutagenesis |
| V420S_Rev | GCATGGACTAATAGGACGCAGCCTGAAGATGACATCCGGGCATTGCTTAGAT | site-directed mutagenesis |
| T479K_For | CAGAGTTAGAGAGAGGTGATACTCCAAAGTCCATCCAATGCTACATGCAAGAC | site-directed mutagenesis |
| T479K_Rev | GTCTTGCATGTAGCATTGGATGGACTTTGGAGTATCACCTCTCTCTAACTCTG | site-directed mutagenesis |
| T479N_For | CAGAGTTAGAGAGAGGTGATACTCCAAATTCCATCCAATGCTACATGCAAGAC | site-directed mutagenesis |
| T479N_Rev | GTCTTGCATGTAGCATTGGATGGAATTTGGAGTATCACCTCTCTCTAACTCTG | site-directed mutagenesis |
| T479S_For | CAGAGTTAGAGAGAGGTGATACTCCATCTTCCATCCAATGCTACATGCAAGAC | site-directed mutagenesis |
| T479S_Rev | GTCTTGCATGTAGCATTGGATGGAAGATGGAGTATCACCTCTCTCTAACTCTG | site-directed mutagenesis |

**Figure S1.** Multiple sequence alignment of the novel IspS Arecaceae proteins with previously characterized IspS from *Arundo donax* and *Pueraria montana*. The amino acids of the diagnostic tetrad are highlighted in orange, while the predicted chloroplast transit peptides are indicated in light blue. Gaps are indicated by dashes, while dots indicate amino acids identical to the first sequence in the alignment.


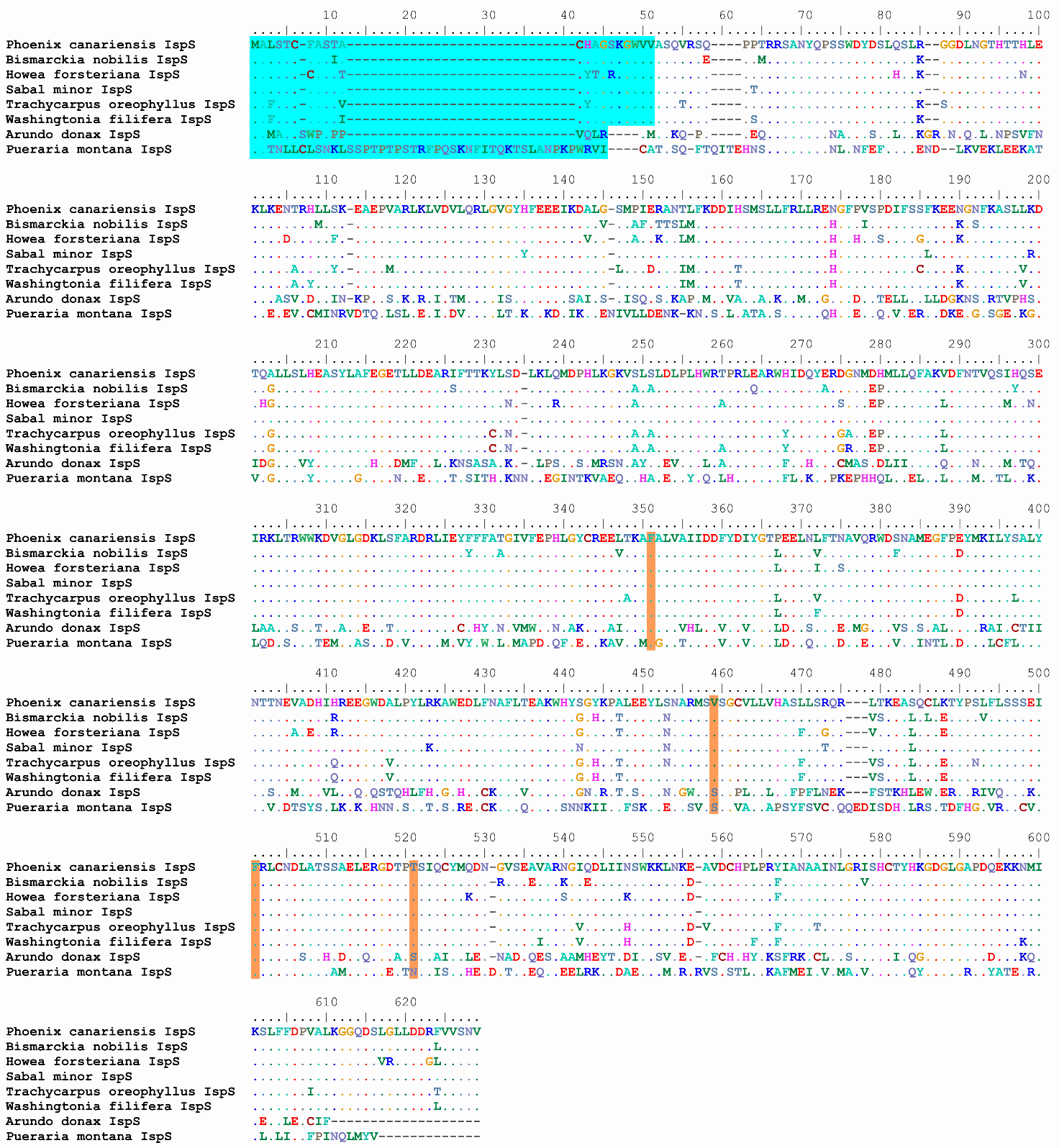

Supplement: Supplementary file 1 — Supplementary Material [file EVA-14-902-s003.docx]
